# Oestrogen receptor positive breast cancer and its embedded mechanism: breast cancer resistance to conventional drugs and related therapies, a review

**DOI:** 10.1098/rsob.230272

**Published:** 2024-06-19

**Authors:** Manu Yadav, Ishita Vaishkiar, Ananya Sharma, Akanksha Shukla, Aradhana Mohan, Madhuri Girdhar, Anil Kumar, Tabarak Malik, Anand Mohan

**Affiliations:** ^1^ Division of Genetics, ICAR- Indian Agricultural Research Institute, Pusa, New Delhi, India; ^2^ Amity Institute of Biotechnology (AIB) University, Amity University Noida, Noida, India; ^3^ Department: Botany and Microbiology, Hemwati Nandan Bahuguna Garhwal University, Srinagar, India; ^4^ School of Bioengineering and Biosciences, Lovely Professional University, Phagwara, Punjab, India; ^5^ Department of Biomedical Engineering, University of Michigan, Ann Arbor, MI, USA; ^6^ Division of Research and Development, Lovely Professional University, Phagwara, Punjab, India; ^7^ Gene Regulation Laboratory, National Institute of Immunology, New Delhi, India; ^8^ Department of Biomedical Sciences, Institute of Health, Jimma University, Jimma, Oromia 378, Ethiopia

**Keywords:** breast cancer, drug-resistance, therapeutic approaches, miRNA alteration, epigenetic alterations, novel target

## Abstract

Traditional medication and alternative therapies have long been used to treat breast cancer. One of the main problems with current treatments is that there is an increase in drug resistance in the cancer cells owing to genetic differences such as mutational changes, epigenetic changes and miRNA (microRNA) alterations such as miR-1246, miR-298, miR-27b and miR-33a, along with epigenetic modifications, such as Histone3 acetylation and CCCTC-Binding Factor (CTCF) hypermethylation for drug resistance in breast cancer cell lines. Certain forms of conventional drug resistance have been linked to genetic changes in genes such as *ABCB1*, *AKT*, *S100A8/A9*, *TAGLN2* and *NPM*. This review aims to explore the current approaches to counter breast cancer, the action mechanism, along with novel therapeutic methods endowing potential drug resistance. The investigation of novel therapeutic approaches sheds light on the phenomenon of drug resistance including genetic variations that impact distinct forms of oestrogen receptor (ER) cancer, genetic changes, epigenetics-reported resistance and their identification in patients. Long-term effective therapy for breast cancer includes selective oestrogen receptor modulators, selective oestrogen receptor degraders and genetic variations, such as mutations in nuclear genes, epigenetic modifications and miRNA alterations in target proteins. Novel research addressing combinational therapies including maytansine, photodynamic therapy, guajadiol, talazoparib, COX2 inhibitors and miRNA 1246 inhibitors have been developed to improve patient survival rates.

## 1. Introduction

**Figure 1.**
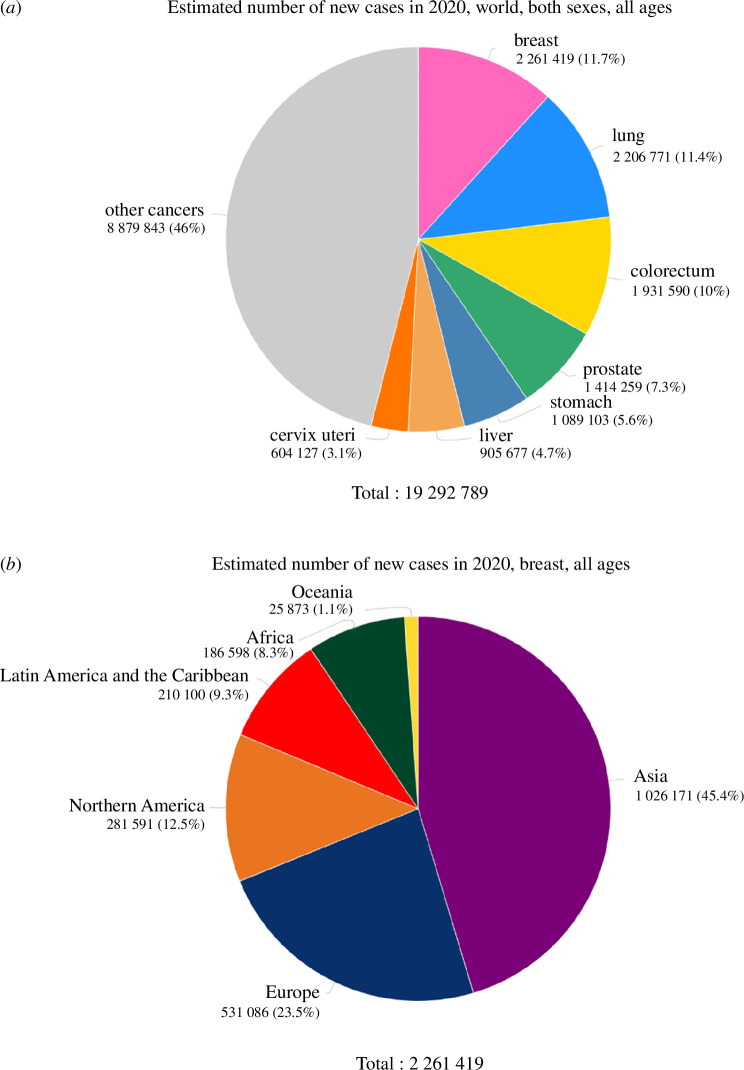
GLOBOCAN data. (*a*) Total breast cancer cases in the world according to 2020 data; (*b*) female population affected by BC [1].

Cancer incidence has increased over the past years. According to the Global Cancer Observatory (GCO), in 2020, overall 19.3 million cases of cancer were reported worldwide, which was an approximately 6.1% increase in the total cancer cases in comparison to 2018 data. It is evident in the 2020 report of GCO, where breast cancer (BC) incidences show a significant percentage of 11.7, the highest among all cancers (figure 1). The same report highlights the highest incidence of this cancer type in females, standing at 24.5% of the total cancers reported in females, mostly occurring among the Asian population. According to these data, women above the age of 50 years are majorly affected.

Oestrogen has a substantial role in the inception and growth of hormone-dependent BC [2]. Studies have repeatedly demonstrated that lifetime oestrogen exposure increases the risk of BC over time [3]. It is further enhanced by persistently prominent levels of oestrogen in the blood [4]. It is detected by clinical markers, such as age at menarche, menopause, alcohol consumption, obesity and although still debatable, exposure to exogenous oestrogen, such as some types of oral contraceptives and hormone therapy [5]. Some of these variables also raise the likelihood that BC will be oestrogen receptor (ER) positive [6]. Studies have shown that higher levels of endogenous oestrogen and testosterone (which are converted to oestrogen by aromatase) increase the risk of BC regardless of the estimated risk of the disease [7].

BC is divided into two groups according to the level of ER in the cancer patient, i.e. ER-positive (ER+) and ER-negative (ER− [8]). Both types have varied characteristics and arise from different kinds of mutation [9]. ER+ BC is detected by various clinical and molecular characteristics. The ER+ BC can be Luminal A type, where the cells are progesterone positive (PR+), cytokeratin8/16+ and human epidermal growth factor-2 negative (HER2-) or luminal B which is characterized as PR+, cytokeratin8/18+ and HER2+ [8,10]. The ER− BC exhibits three types—HER2-enriched type, basal-like and normal-like [8]. HER-2-enriched is characterized by only HER2+ receptors and ER as well as lacking PR. In basal-like, all the hormone receptors (ER, PR, HER2) are absent and cytokeratin 5/6, 14 and 17 as well as epidermal growth factor (EGF) is positive. In normal-like BC, all the molecular indicators are absent [10].

Out of the two—ER+ and ER− BC, ER+ BC is more prevalent in the world. An important treatment for ER+ BC is endocrine therapy. This therapy is found to be beneficial except in situations of illness that are urgently life-threatening, such as a visceral crisis, it becomes the therapy of choice in the metastatic scenario [11,12]. Traditional endocrine treatments, such as selective ER modulators (SERMs) and aromatase inhibitors (AIs) letrozole, anastrozole and exemestane, try to lower oestrogen levels or regulate oestrogen action by inhibiting the ER [13]. In post-menopausal women, the main sites where oestrogen biosynthesis occurs are skin, muscle, fat and benign and malignant breast tissue [14]. AIs should only be used in post-menopausal women, as opposed to SERMs such as tamoxifen, which can be used for women in either the pre- or post-menopausal phase. Tamoxifen is effective in pre-menopausal patients as this group of patients actively have oestrogen-secreting ovaries. In post-menopausal patients, oestrogen is produced by the conversion of androgen with the help of the aromatase enzyme present in peripheral tissues, hence AI is effective in such patients as it hinders the action of aromatase. So, before using AIs in pre-menopausal women, it is necessary to undergo ovarian suppression, and currently, this is often accomplished by giving Gonadotropin releasing hormone (GnRH) analogues [15].

Greater knowledge of the function of oestrogen in BC has resulted in treatment approaches that target enhancing the function of the ER and its intracellular signalling pathway. Oestrogen plays a role in the development and progression of BC. In its penultimate stage, oestrogen production is catalysed by the enzyme aromatase, which has been identified as a target suitable for selective suppression. The modern third-generation AIs efficiently suppress oestrogen synthesis while having no negative effects on other steroidogenic pathways [16].

The effectiveness of contemporary endocrine therapy remains constrained by either inherent or acquired resistance, even though it has significantly improved outcomes for women with ER+ BC. Tamoxifen, a SERM, functions as an anti-oestrogen by preventing the binding of ERs to oestrogen [17,18]. After 2–5 years of tamoxifen therapy, one-third of women with early-stage BC may develop drug resistance [19]. The risk of endometrial cancer may increase by twofold in post-menopausal women who use tamoxifen over time. As a result, tamoxifen is frequently supplemented or replaced with AIs and fulvestrant (which is an anti-oestrogen antagonist) as a second, and increasingly first-line, endocrine treatment. In comparison to tamoxifen, AIs appear to have improved anti-tumour activity, and more crucially, they may be helpful for tamoxifen-resistant patients, according to several clinical trials [20]. However, in a study, several imidazole anti-mycotic medications were discovered to be effective in suppressing aromatase but not in anti-cancer activity in the case of BC. Ketoconazole, miconazole and clotrimazole were discovered to have greater inhibitory activity than aminoglutethimide, however, they are not employed for anti-cancer activity [21]. When studied further, fulvestrant alone or in combination with letrozole has shown great potential for post-menopausal women to postpone the onset of acquired resistance. Resistance to fulvestrant and AIs develops gradually, even in cases when the treatment is initially effective [22].

Therefore, access to proper medication administration by responsive individuals requires knowledge of the underlying mechanisms of the drug action, factors contributing to it and resistance to these drugs.

## 2. ER-related Drugs

**Figure 2.**
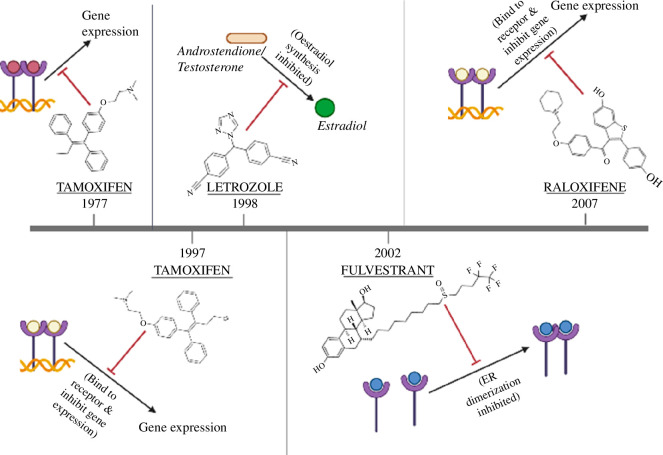
ER-related drug FDA approval timeline and mechanism. FDA, Food and Drug Administration.

Since oestrogen is a steroidal hormone, ER+ cancers can be prevented and treated with approaches that either involve drugs that block the receptor or block the production of oestrogen (figure 2). These drugs, also termed anti-oestrogen, are hormone antagonists that block oestrogen and suppress its production [23]. Anti-oestrogens include selective ER modulators such as tamoxifen, raloxifene, toremifene and selective oestrogen receptor degraders (SERD) like fulvestrant; table 1 summarizes five conventional drugs used in BC treatment. Tamoxifen, toremifene and Rrloxifene are SERM acting as antagonists by inhibiting the binding of oestrogen to ER. Their effect may vary like arresting the cell cycle at the G1 phase, DNA damage reduction and another anti-oestrogenic effect that ultimately causes tumour growth suppression. Meanwhile, fulvestrant is another drug belonging to the SERD category that hinders ER dimerization and letrozole is an AI that inhibits aromatase enzymatic activity.

**Table 1.** Details of various ER drugs, their type and action.

no.	ER^a^-related drug	type	action	effect	references
1.	Tamoxifen	SERM^b^	act as an antagonist by inhibiting oestrogen binding to ER	arrest cell cycle at G1 phase and decrease cell growth	[17,24–26]
2.	Toremifene	SERM	act as an antagonist by inhibiting oestrogen binding to ER	reduced oestrogenic activity and DNA damage. development of anti-tumour adducts in endometrium	[27–29]
3.	Raloxifene	SERM	agonist as well as antagonist effect on ER receptor	may have oestrogenic actions on bone and lipid metabolism anti-oestrogenic actions in the breast a neutral effect on the endometrium	[30–33]
4.	Fulvestrant	SERD^c^	inhibit ER dimerization by inactivating AF1^d^ and AF2	decreased localization of receptor to nucleus and hence inhibition of oestrogen-dependent gene expression	[34–36]
5.	Letrozole	AI	aromatization by inhibiting the enzymatic activity of intracellular aromatase at the primary locations	oestrogen production suppressed	[37,38]

^a^ Estrogen receptor.

^b^ Selective ER modulators.

^c^ Selective ER degrader.

^d^ Activating functional domain 1 and 2.

### 2.1. Tamoxifen

In 1977, the Food and Drug Administration (FDA) approved tamoxifen for the treatment of women with metastatic BC, and a few years later for the adjuvant therapy of initial BC [39]. Tamoxifen, a selective SERM, inhibits the E2-mediated action of AF2, causing it to become ER-antagonistic while still retaining some partial agonistic effect [17]. Tamoxifen functions as an anti-oestrogen by preventing the binding of ERs to oestrogen. The anti-oestrogenic properties of tamoxifen are assumed to be responsible for its anti-cancer actions, which are mediated via competitive suppression of oestrogen binding to ERs [18]. The inhibition of oestrogen-regulated genes by tamoxifen includes growth factors and angiogenic factors that may stimulate growth by paracrine or autocrine mechanisms. This causes a block in the cell cycle’s G1 phase and a slow down of cell growth.

Further, tumours could recede as a result of the shifted equilibrium between cell growth and persistent or programmed cell death [24,25]. Tamoxifen reportedly inhibits oestrogen from initiating the secretion of either transforming growth factor-beta (TGF-β ) or EGF from BC cells to inhibit tumour growth as observed in *in vitro* studies [40].

### 2.2. Toremifene

For the treatment of metastatic BC in post-menopausal women with ER+ or ER undetermined tumours, toremifene is recommended [41]. Its structure differs from tamoxifen, which was the most popular and oldest selective ER modulator, used in endocrine treatment. However, after long-term tamoxifen administration, several serious side effects were reported. Hence, toremifene, a chlorinated derivative of tamoxifen, where one of the hydrogen atoms in the ethyl side chain is replaced with a chlorine atom [42], was developed in 1981 with the goal of having similar efficacy to tamoxifen and a safer profile. Also, toremifene showed better results in patients with the CYP2D6 mutation as well as in patients suffering from fatty liver and liver fibrosis [43,44]. It was approved by the FDA in 1997 for ER+ post-menopausal women [45]. It has been demonstrated in pre-clinical studies that toremifene has similar ER binding and reduced oestrogenic activity and DNA damage with effective development of anti-tumour adducts in the endometrium [27,28]. It is a non-steroidal, selective ER modulator made of triphenylethylene. Depending on the dosage, length of the therapy, the target organ or the endpoint employed, toremifene binds to the ER with either oestrogenic or anti-oestrogenic qualities [46,47]. In 2018, a Chinese study reported the considerable benefit of toremifene experienced by CYP2D6*10 T/T patients, who made up one-fifth of the sample population. In this class of patients, toremifene may be a viable alternative for adjuvant endocrine treatment in China [27].

### 2.3. Raloxifene

The non-steroidal benzothiophene family of drugs includes raloxifene, an oral SERM of the second generation. Raloxifene displays partial agonist and antagonist action in pre-clinical investigations, depending on the tissue [30]. To either stimulate or suppress endogenous oestrogen effects, raloxifene competes with them for binding to the ER. Raloxifene’s agonistic or antagonistic effects rely on the co-activators and co-repressors that are attracted to the ER target gene. The molecular mechanisms behind the tissue-specific pharmacologic effects of raloxifene have previously been poorly understood. The medicine has oestrogenic effects on bone and lipid metabolism, anti-oestrogenic effects in the breast and a neutral impact on the endometrium, which highlights its intriguing pharmacology [31,32]. Early in the trial, raloxifene failed the effectiveness test. Early research on post-menopausal women with metastatic BC that was initially ER+ did not demonstrate effective treatment [48]. Research showed that raloxifene was effective in preventing invasive BC, the USFDA approved it for use in lowering BC risk in 2007. Eventually, however, the STAR (Study of Tamoxifen and Raloxifene) trial assessed the efficacy of raloxifene and tamoxifen in preventing BC in high-risk women [49,50].

### 2.4. Fulvestrant

Fulvestrant, unlike SERMs, does not resemble oestrogen. It is referred to as a pure anti-oestrogen for this reason. It targets the ER for degradation when it binds to it and destroys it [17]. Tamoxifen, also a partial agonist of ER, functions nearly entirely as an ER antagonist [51]. It is a widely used drug for ER+ patients owing to its antagonist properties. But in the long term, it exhibits agonist properties in other organs like endometrial proliferation in menopausal women. So, an ER downregulator, fulvestrant was discovered, which binds to monomers of ERs inhibiting its dimerization [52]. It is 7*α*-alkylsulphinyl that is an analogue of E2 and shows pure antagonist activity [53]. It inactivates AF1 and AF2 causing decreased localization of receptors to the nucleus and hence inhibition of oestrogen-dependent gene expression. The complex formed by ER and fulvestrant is unstable and is degraded at an accelerated rate [53]. It is an important novel therapy as its mode of action is not cross-resistant to existing therapies [52].

Fulvestrant, in contrast to tamoxifen, changes the shape of the ER and obstructs both AF2- and AF1-related transcriptional activity [34]. Additionally, the unstable complex that fulvestrant generates when it binds to the ER contributes to its rapid breakdown. Because of its dual roles as a competitive antagonist and a SERD, fulvestrant lowers the levels of cellular ER alpha [35]. A study in Spanish ER+ BC patients revealed that fulvestrant when compared with a currently used drug, anastrozole, was much more effective [54]. A Chinese population study in ER+ menopause patients also showed that patients treated with fulvestrant had longer overall survival (OS) than the patients treated with anastrozole [55]. A Canadian study revealed better OS in treatments where chemotherapy was followed by fulvestrant [56], which was approved by the FDA in the 2002.

### 2.5. Letrozole

Letrozole reduces the amount of oestrogen synthesized in all tissues by competitively binding to the cytochrome P450 subunit of the enzyme aromatase to inhibit it. It successfully inhibits the synthesis of oestrogen, which prevents the growth or induces the regression of hormone-responsive breast tumours *in vivo*. Letrozole is a more potent AI, according to randomized controlled research, comparing the effects of letrozole and anastrozole on oestrogen levels. Tamoxifen is still useful even when AI is not permitted [57]. While compounds containing nitrogen, such as imidazole and triazoles, bind to the iron in CYP-450’s heme moiety, letrozole’s cyanobenzyl moiety resembles the steroid backbone of the enzyme’s natural substrate, androstenedione. Additionally, it was discovered that letrozole, a triazole molecule, inhibits aromatase *in vivo* better than other fadrozole analogues [37], which was approved by the FDA in 1998 [58].

## 3. Drug resistance in breast cancer and its types

Up to 90% of BC deaths are caused by drug-resistant BC. The current cancer treatments become less effective, which frequently results in metastasis and recurrence. About half of BC patients with drug resistance had both inherited and acquired resistance [59]. Genetic mutations, activation of certain innate pathways which develop prior to therapy, can all lead to intrinsic resistance [60].

## 4. Genes, miRNA and epigenetics reported resistance in breast cancer

**Figure 3.**
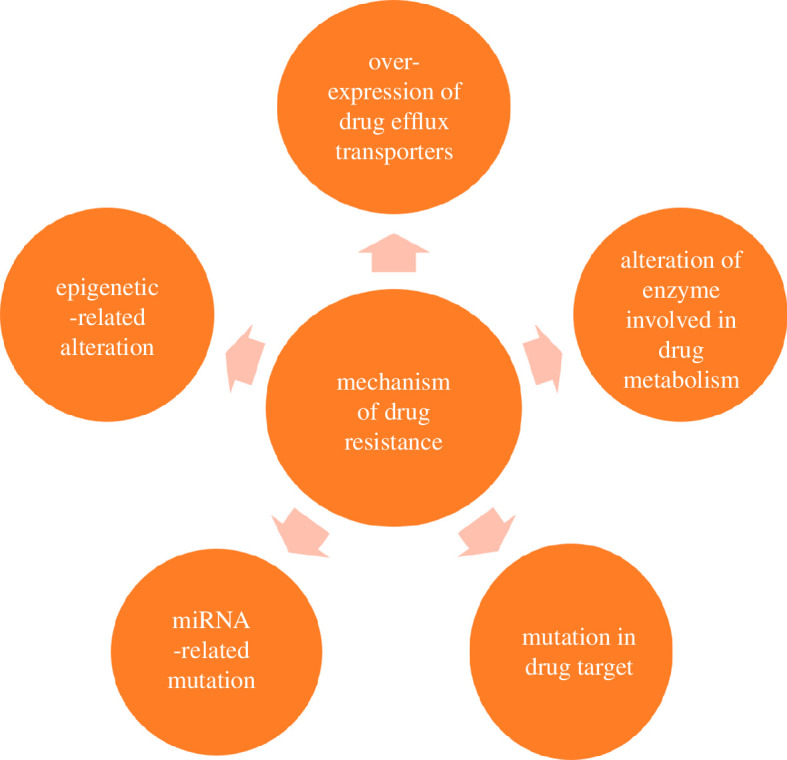
Illustrates the ways in which drug-resistance in BC arises.

There have been cases where the drugs above were resisted, and so the cancer then got categorized as drug-resistant BC. It occurs owing to the changes in proteins present in the cells. The structural functional changes in these proteins arise owing to mutational or epigenetic variations in the gene. It may also occur owing to variations in miRNA levels within the cells; figure 3 illustrates five major mechanisms of such alterations and variations, including overexpression of drug efflux transporters, alteration of enzymes involved in drug metabolism, drug target mutations, miRNA expressional alterations and epigenetic alterations [61–69].

### 4.1. Breast cancer resistance owing to genetic alterations

One of the major contributors of drug resistance in BC is genetic alteration. Genes undergo transcription and translation to form proteins and the expression of these genes is regulated by numerous factors. The alteration in the regular transcriptional process further results in variation in the proteins translated by the genes. These variations could be at the levels of synthesis of these proteins and could also involve complete inhibition of the production of proteins that play an incredibly significant role in cell growth and the execution of regular cellular processes. Additionally, the altered gene expression can also lead to the production of proteins that are normally not produced in a healthy body and could be disease causing in several ways. As the normally expressed cellular proteins participate in various signalling pathways of the cell, the alteration in the concentration of protein can potentially alter these pathways, which eventually helps in the process of growth, invasion as well as metastasis of cancerous cells. This may be owing to the mutational changes in genes that also result in changes in the protein structurally as well as functionally. These mutations can lead to changes in receptors, immune response-related proteins or proteins involved in the signalling cascade.

ATP-binding cassette (ABC) transporters are among the protein structures whose gene polymorphism results in the occurrence of drug resistance in the case of BC [70]. These proteins normally act in cholesterol homeostasis, immune recognition as well as drug efflux [71]. ABC is a transporter molecule involved in various drug delivery mechanisms, especially chemotherapeutic drugs [72,73]. Majorly, TM3, TM8 and TM9 are the transmembrane domains subjected to high mutation [74]. It is further grouped into ABCB, ABCCI, ABCCII, ABCD, ABCF and ABCG. ABCBI also known as the multi-resistance gene encodes for permeability-glycoprotein (P-gp). P-gp is responsible for the movement of drugs out of the cell with the use of ATP in the process. The polymorphism of the *ABCB1* gene at 2677 position has been observed to be involved in paclitaxel and anthracycline resistance [75,76]. In addition, COX2 engages in promoting cancer cell properties. It also upregulates efflux transporters like P-gp resulting in multiple drug resistance (MDR [77]). Also, in the Asian population, the *ABCC1* mutation results in betulin resistance owing to overexpression of *ABCC1* [78]. Even *ABCG2* variations have been shown to increase drug efflux owing to the overexpression of the *ABCG2* gene [61,62]. The variation causes BC to become resistant to tyrosine kinase inhibitors, doxorubicin as well as mitoxantrone [79]. In recent studies, it has been observed that *ABCG2* overexpression also causes tivantinib resistance [80].

AKT, a ser/thr kinase is one of the proteins participating in the PI3K (phosphatidylinositol 3-kinase)/AKT (protein kinase B)/mTOR (mammalian target of rapamycin) signalling pathway. The pathway majorly participates in cell glucose metabolism, DNA repair, proliferation, apoptosis as well as survival [63,64]. Other than the PI3K/AKT/mTOR pathway, AKT also interacts with Notch, Nuclear factor kappa B (NF-κB), Snt/β-catenin and Notch to initiate other signalling pathways [81]. AKT interaction with Mitogen-activated protein (MAP) kinase results in a change in PD-L1 expression, while interaction with Notch and β-catenin affects cell migration as well as the integrity of cancerous cells. In hypoxic conditions, AKT increases the concentration of mitochondria in cancerous cells and alters mitochondrial metabolism suppressing the apoptosis process. This mechanism of AKT overactivation has been reported in paclitaxel resistance, used to treat triple-negative breast cancer (TNBC) [81]. In the study by Chung do Gao *et al.*, the AKT1 polymorphism-rs121434592 (*E17K*) proved to be a contributor to the proliferation of cancerous cells present in the breast and may cause resistance to endocrine therapy [82–84].

**Figure 4.**
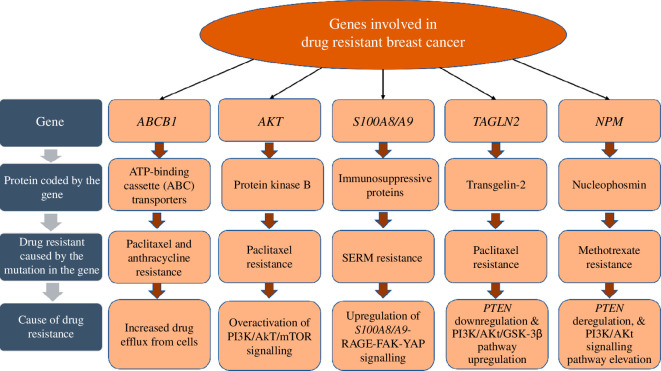
Genes involved in drug-resistant BC and their effect.

Also, high expression of *S100A8* and *S100A9* genes has been researched to be a reason for the downregulation of *ESR1* [65]. *S100A8* as well as *S100A9* proteins are released by the suppressor cells derived from myeloid cells which are responsible for the immunosuppressive function of cancerous cells [65,85]. This may be a cause of developing resistance in breast cancerous cells to the drugs targeting the ERα receptor, i.e. SERMs [86,87]. Other than an ER-related function, in TNBC, *S100A8* and *S100A9* are upregulated along with the Receptor for Advanced Glycation Endproducts (RAGE) protein. S100A8 and S100A9 activate RAGE which phosphorylates Focal Adhesion Kinase (*FAK*) and fatty acid formation. These events result in the accumulation of Yes-Associated Protein1 (*YAP*) in the nucleus, hence, gene transcription. The *S100A8/A9*-RAGE-FAK-YAP signalling induces TNBC cell proliferation and migration [88]. *S100A8* upregulation causes tamoxifen resistance [89]. Figure 4 illustrates the five genes discussed that are the cause of drug-resistant properties of BC. *ABCB1* encodes for ABC transporters, which in mutation increases drug efflux causing paclitaxel and anthracycline resistance. AKT is responsible for the transcription of a protein kinase B, whose mutation causes overexpression of the PI3K/AKT/mTOR signalling pathway leading to paclitaxel resistance. Some immunosuppressive proteins are encoded by S100A8/A9 which on alteration is upregulated, and in turn, upregulates RAGE-FAK-YAP signalling and causes SERM drug resistance. Another signalling PI3K/Akt/GSK is also upregulated by *TAGLN2* and *NPM* polymorphism resulting in paclitaxel resistance and methotrexate resistance, respectively [70,81,89–91].

*TAGLN2* **,** was researched by Liu *et al*. recently as a potent gene contributing to paclitaxel resistance among BC patients [90]. The gene encodes for transgelin-2 which is an actin binding protein involved in the actin-associated signalling pathway [92]. The overexpression of transgelin-2 in MCF-7/S cells resulted in the increase of ABC transporters while leading to a reduction of *PTEN* expression [90]. *PTEN* codes for a tumour suppressor enzyme and is involved in the dephosphorylation of phosphatidylinositol (3,4,5)- triphosphate, thus, negatively regulating the PI3K/AKT pathway [93]. *PTEN* downregulation leads to the upregulation of the PI3K/Akt/GSK-3β pathway. This promotes both the migration as well as the invasive property of cancer cells. As paclitaxel targets PTEN expression, upregulating it [91], transgelin-2 mediated alteration results in paclitaxel resistance [90]. Nucleophosmin (*NPM*) has also been shown to regulate *PTEN* expression. MCF-7/MTX (methotrexate-resistant BC cell line) cells exhibited elevated levels of *NPM* expression. This deregulated *PTEN* leads to an elevation in the PI3K/Akt signalling pathway [91,94].

Maytansine, an anti-tumour agent, as well as its artificial variations at very low molecular concentrations, has also been studied to kill cancer cells by preventing mitosis and causing cytotoxicity. Maytansine derivatives were created in order to address the systemic side effects of the drug. These derivatives were then covalently coupled with antibodies specific to tumour-associated antigens, to enable targeted drug administration and cancer cell death. T-DM1, a maytansine-antibody combination with FDA approval, is used to specifically target HER+ BC [95]. Furthermore, the abnormal glycolytic phenotype of cancer cells has been linked to mutations on nuclear genes that affect the activity of succinate dehydrogenase [96], a part of the respiratory chain and the Krebs cycle, and thus participate in mitochondrial energy transduction. These mutations have also been linked to the accumulation of a metabolic intermediate (succinate) that inhibits HIF-1α prolyl-hydroxylase. The glycolytic shift of BCs is invariably associated with a profound alteration of the mitochondrial proteome of the BC cell, as revealed by Fisher’s and cluster analysis; these findings are consistent with previous findings in other human cancers, and thus provide support for the theory that an impaired mitochondrial function also causes the glycolytic shift of BCs [97].

### 4.2. miRNA reported in breast cancer

MicroRNA (miRNA) are non-coding RNAs with a small length of 21–25 nucleotides responsible for the regulation of gene expression. The proliferation as well as metastasis of BC have been found to be related to abnormal miRNA expression [66,67]. Alteration in miRNA expression has also proved to be a reason for the occurrence of drug resistance in BC, such as miRNA-33a, miRNA-27b, 298, 1246 and many more. miR-33a is one of the miRNAs reported in BC drug resistance. It inhibits gene *ABCA1* expression and also hinders cholesterol reverse transport in High-density Lipoprotein (HDL) [67,98].

miR-33a activates AMPKα which further activates the transcription of genes responsible for ATP production [99]. This miRNA expression has been shown to cause fulvestrant resistance in the cell line of triple-negative breast cancer (TNBC) [98]. All these changes cause radiation-resistant BC [67]. *miRNA27b* has proved to be a tumour suppressor and also helps in enhancing the efficiency of anti-cancer drugs by promoting activation of the p53 apoptosis pathway and also deregulating the drug detoxification pathway in the therapy of kidney and liver cancer [100,101]. However, miRNAs function diversely in different cancers. Contrary to its function in liver and kidney cancer, *miRNA27b* is involved in cancer progression as well as drug resistance of BC [102,103]. Its upregulation activates NF-kB signalling leading to nischarin resistance [102]. The lower concentration of *miRNA27b* as well as *miRNA451a* lowers TAC (combination of Taxotere, Adriamycin and cyclophosphamide) chemotherapy response [104].

*miR-298* is also involved in doxorubicin resistance [105]. MDA-MB-231 is a cell line of TNBC cells that are resistant to chemotherapy and was used to study doxorubicin resistance. Cells resistant to the drug showed varied expression of various miRNAs. Doxorubicin resistance is owing to the high expression of P-gp (ABC transporter) mRNA, which increases the drug efflux in cells. Here, the miR-298 level was observed to be involved in P-gp mRNA expression. Doxorubicin-resistant cells in the MDA-MB-231 cell line showed lower levels of miR-298. The low level or absence of miR-298 resulted in high expression of P-gp mRNA owing to the absence of dicer [106].

In serum samples, *miR-1246* is seen to be elevated in BC patients. Normally, epithelial cells release miR-1246 in the mammary gland which gets stored in tissue. When these cells turn malignant, miR-1246 enters the blood circulation, which is observed in patient serum samples [107]. miR-1246 directly targets cyclin G2 (*CCNG2*) which is a tumour suppressor gene [108]. Elevation of *miR-1246* reduces the *CCNG2* concentration in BC [109]. miR-1246 binds to 3’-UTR of CCNG2, enhancing its expression. The genetic overexpression in the cells of the MDA-MB-231 cell line leads to the activation of the NF-κB signalling pathway that mediates cell growth as well as invasion [108,110].

Other than these, *miRNA-3130* as well as *miRNA-584* were upregulated and downregulated, respectively, in one of the multi-resistant drug cell lines, Bads-200. Even in Bats72 cells, other multi-resistant cell lines, *miR-92a* and *miR-4277* levels are elevated. This research would be helpful in the therapy of neoadjuvant chemotherapy-resistant BC [111]. Also, a potent therapy researched to overcome doxorubicin resistance in BC is inhibiting lncRNA-HOTAIR. The long-noncoding RNA hinders the phosphorylation of AKT, PI3K and mTOR which leads to the activation of PI3K/AKT/mTOR signalling that contributes to the proliferation of cells. Additionally, lncRNA acts on Bcl-2, Bax and Caspase-3 expression levels leading to the inactivation of apoptosis signalling in cancerous cells [112].

### 4.3. Epigenetics reported in breast cancer

Epigenetic changes are required to assist gene expression in cells. Alteration in the epigenetic mechanism may cause faulty expression of the genes which may contribute to cancer development.

**Figure 5.**
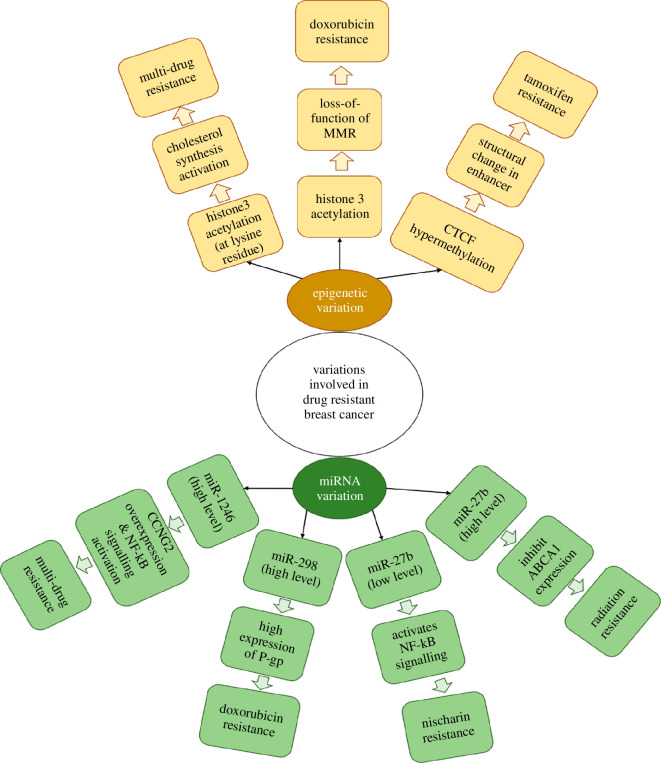
Illustrations of epigenetic and miRNA variation involved in drug-resistant BC.

*DOK7* hypermethylation is detected in BC cells resistant to tamoxifen [68,113]. *DOK7* functions as a substrate that eventually participates in tyrosine kinase activation. This supports proliferation as well as the survival of BC cells [114]. The hypermethylation at CCTC-Binding factor sites has been shown to cause changes in enhancer chromatin structure leading to loss of tamoxifen binding to ER causing depleted interaction of enhancer to promotor [115]. The *NCOR2* gene shows metastasis-free survival in ESR1 BC patients [116]. However, methylation of the *NCOR2* enhancer results in poor prognosis for patients [117]. A low level of ERα production was observed owing to ESR1 promoter methylation. This epigenetic change has been reported in tamoxifen resistance [118]. Figure 5 illustrates miRNA and epigenetic variation causing drug-resistant BC. *miR-33a*, *miR-298* and *miR-1246* cause radiation resistance, doxorubicin and multi-drug resistance, respectively. Also, miR-27b downregulation leads to nischarin resistance. Epigenetic variations like CTCF hypermethylation and histone3 acetylation have been observed to show tamoxifen and doxorubicin resistance, respectively [67,102,104,106,108,110,111].

Acetylation of histone3, i.e. H3K56ac, H3K18ac, H3K27ac and also its methylation, i.e. H3K4me3, owing to increased concentration of *HAT1* and *HMT1* leads to doxorubicin-resistant BC. Hypermethylation has been observed at the promotor of MSR2 domain in BC cells resistant to doxorubicin [69]. This epigenetic change causes the loss of function of DNA mismatch repair (MMR) system that helps in DNA repair, induction of apoptosis, as well as DNA damage signalling [119,120]. The loss of MMR protein results in the halting of chemotherapy-induced caspases involved in apoptotic signalling [121].

Long-term exposure to therapies such as endocrine therapies results in epigenetic changes in the cells [122], such as exposure to AIs causing lysine acetylation at histone3. The acetylation results in the activation of enzyme transcription involved in cholesterol synthesis. This increases 27-hydroxylcholestrol concentration which, in turn, increases chromatin binding of ERα [116,117].

## 5. Detection of alteration in BC patients

In order to provide patients with personalized medication/therapy, it is necessary to develop an accurate and precise detection methodology. There are various methods which can be, or are being, used to detect mutations and other variations.

### 5.1. Digital Polymerase Chain Reaction (PCR)

The most common methodology that is widely used is Polymerase Chain Reaction (PCR). Digital PCR (dPCR) is a more precise method that helps to detect single positives by dividing the sample into a large number of individual reactions. Two methodologies are used in this technique—chip dPCR (cdPCR) and droplet dPCR (ddPCR). The cdPCR processes the sample by dividing it into a nanolitre reaction chamber and provides results by detecting fluorescence using an imaging system along with inverted endoscopy. ddPCR processes the sample by creating a microlitre reaction using water-in-oil as well as microfluid technology and the results are given by reading the signals using two coloured detectors [123]. The method was used to detect results in S100A4-mutated ovarian cancer patients [124].

### 5.2. Next generation sequencing

Genetic alteration can be detected in BC patients by various methods. Next-generation sequencing (NGS) is one of the techniques that can be used to detect mutations when combined with protein expression profiling [125]. Tumour cells release various components into the circulatory system, like circular tumour DNA, cell-free RNA, circulating tumour cells, tumour-educated exosomes and platelets [126]. The NGS ctDNA (circular tumour DNA) platform is a viable novel option to detect genetic alterations as well as allelic frequency in patient samples [127]. Also, in a study conducted by Martinez-Gutierrez *et al*., miRNA profiling of metastatic BC patients was done using NGS [128]. It can be used to detect genetic variation, miRNA alterations as well as epigenetic modification.

### 5.3. Chromatin immunoprecipitation

Chromatin immunoprecipitation (ChIP) is a method used to study epigenetic variations, especially on histone proteins. It uses antibodies to precipitate specific proteins and further detect these using various detection techniques such as PCR or NGS [129,130]. However, this method has insufficient coverage in detecting various modifications especially in tumours. So, Grosselin *et al*., developed a high-throughput single-cell ChIP-seq to study the chromatin landscape of thousands of cells at a resolution of a single cell enabling the study of 10 000 loci/cell. In the study, they were able to build and distinguish between the chromatin landscape of stomal and tumour cells detecting H3K27me3 in drug-resistant tumour cells [131].

### 5.4. Electrochemical biosensor

Pothipor *et al*. developed an electrochemical biosensor claiming to be a potential BC diagnosis method. The biosensor is based on a gold nanoparticles/graphene quantum dots/graphene oxide (AuNPs/GQDs/GO) modified three-screen-printed carbon electrode (3SPCE) array. The multiplex biosensor uses three redox species (AQ, MB and PDA) connected to the AuNPs/GQDs/GO-modified 3SPCE as distinct signals, and the AuNPs/GQDs/GO composite serves as a signal enhancer. The method is overly sensitive and has multiplexing capability. It has been shown to be a great method to detect miRNA-21, miRNA-155 and miRNA-210 in BC patients [132].

## 6. Therapeutic methods to overcome drug resistance

The mechanisms involved in tumour development are being explored to find novel diagnostic as well as therapeutic targets. These therapies target the genes altered in BC drug resistance. Photodynamic therapy (PDT) is one of the techniques researched to be a potent method to cure MDR. Encapsulated anti-cancer drugs in nanoparticles when delivered to patients’ bodies, have a high affinity to sugars, proteins, transferrin, folate, aptamers or lipids. These biomolecules are overexpressed in cancerous cells [133]. Nanoparticles enter the cells by the process of endocytosis and also evade the efflux mechanism of ABC transporters once they enter the cytosol [105,134]. Recently, Hong *et al.* used a novel nanocarrier loaded with a photosensitizer drug delivery method to avoid efflux transporters and increase intracellular accumulation. This complex mediates reactive oxygen species production by accumulating in lysosomes which leads to a reduction in ABC transporter expression, hence, increasing the therapy effectiveness [135,136]. Though some past studies have shown lower efficacy of PDT owing to hypoxia conditions along with the increased level of glutathione in tumour cells, Gao *et al.* developed a novel methodology named self-delivery nanorods (AINRs) to overcome the drawbacks. The AINR uses a combined interaction of atovaquone (mitochondrial complex III inhibitors), indocyanine (photosensitizer) and DSPE-PEG 2000 (dispersion stabilizer). *In vitro* experiments on MDA-MB-231 cells and 4T1 cells showed the treatment blocked oxidative phosphorylation in the cancerous cells by mitochondrial complex III inhibition which results in lowering the ATP concentration. The decrease in ATP causes glutathione synthesis inhibition leading to cell cycle arrest at S-phase that enhances PDT efficacy. The methodology has been analysed to be a great way to make PDT successful in TNBC patients [137]. Also, RGD-conjugated RSP (phthalocyanine), a targeted PDT claimed to be an effective way to treat TNBC. The combination results in increased photosensitizer in cancerous cells of the 4T1 cell line (constituting TNBC cells). The therapy showed better results in *in vitro* research as RGD bound to overexpressing ανβ3 integrin receptors resulting in targeted suppression of tumour in TNBC cells [138]. PDT is predicted to show minimal side effects, like, burns, itching, swelling and pain [139].

Other than photosensitizers, guajadial may be a potent drug to overcome drug-resistant BC. In the Yaxun *et al*. study, guajadial which is a meroterpenoid, used in MCF-7/ADR and MCF-7/PTX cell lines (drug-resistant BC cell lines) showed an inhibiting effect on ABC transporters, hence leading to inhibition of drug resistance. Simultaneously, it also inhibited the PI3K/AKT pathway in the cell lines suppressing cell proliferation, apoptosis and migration [140].

Talazoparib is also a novel potent drug that targets ABCG2 of multi-drug resistant cells. The drug downregulates ABCG2 expression by acting on its mRNA synthesis and may provide a cure [141]. Also, the COX2 inhibitor has been observed to be a potential drug to treat patients with an ABC mutation especially in P-gp. The drug might cause fatigue, nausea and anaemia [142,143]. In the study by Zhang *et al*., it was concluded that a combination of COX2 inhibitor and doxorubicin can be used to overcome MDR. In the experiment, for effective co-loading, targeted distribution and controlled release of DOX and CXB, they created HPPDC nanoparticles with hydrophilic hyaluronic acid (HA) shells and hydrophobic disulfide-containing poly (β-amino ester) (ssPBAE)/ poly (lactic-co-glycolic acid) (PLGA) cores. The experiment showed a positive response in overcoming MDR at the cellular as well as animal level when applied using different mechanisms like intercellular drug release and CD44-mediated cellular internalization. This might cause drastic weight loss as observed in the mouse model owing to the absence of tumour-targeting specificity [83,144].

Chen *et al*. studied the effect of FA-17 (synthetic histone deacetylase inhibitor) on MCF-7/MTX. The FA-17 treatment has been shown to suppress the NPM level and PI3K/AKT pathway. This led to the initiation of apoptotic pathways in cancerous cells. Thus, the synthetic histone deacetylase inhibitor proves to be a potent therapeutic method for treating drug-resistant BC [145].

**Figure 6.**
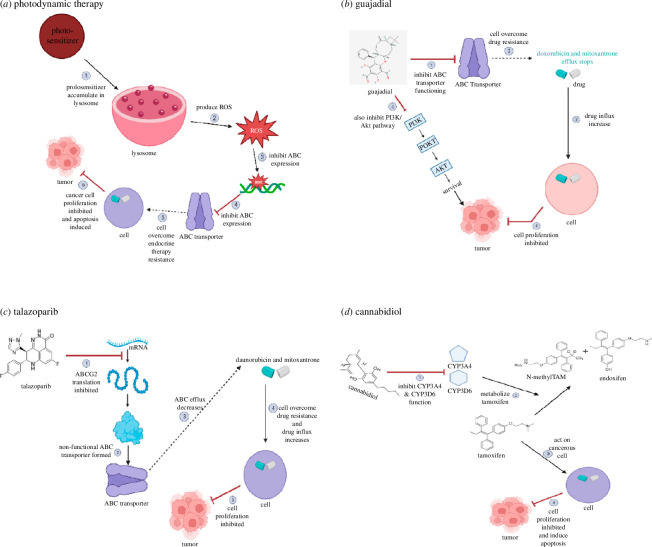
Mechanisms of novel treatments. (*a*) Photodynamic therapy [133,137]; (*b*) Guajadial [140]; (*c*) Talazoparib [141]; (*d*) Cannabidiol [148,149].

For miR-1246 drug resistance, Yue-chu *et al*. designed a miR-1246 inhibitor which was transfected in MDA-MB-231 cells. The inhibitor activated intrinsic along with extrinsic apoptotic pathways by the cleavage of caspase 8 and 9. This resulted in the reversal of docetaxel resistance in the cancerous cells. The other pro-apoptotic proteins like Bcl2, Mcl1, Bad, Bak and many more were also activated. This also inhibited the invasion and migration of MDA-MB-231 cells [146,147]. Figure 6 demonstrates various mechanisms of novel therapy involved in BC treatment.

For CYP2D6-mediated resistance, Parihar *et al*. studied the cannabidiol effect on patient samples. The drug was given along with tamoxifen, this treatment resulted in the detection of the increased level of N-desmethyltamoxifen and endoxifen which are metabolic products of CYP3A4 and CYP2D6, respectively. Analysing this result, Parihar *et al*., concluded cannabidiol is a potent therapeutic inhibitor of CYP3A4 and CYP2D6 [148]. Also, Ahmed and Wober researched a novel triphenylethylene prodrug and found it to be more efficient than tamoxifen in MCF-7 cells. These were reported to be metabolized by carboxylesterases rather than CYP2D6, making them a potent drug for patients with CYP2D6 polymorphism [149]. Cannabidiol does not show major adverse effects other than dizziness and disorientation [26].

Nafamostat mesylate (NM) according to the research conducted by Lin *et al.* successfully resulted in treating tamoxifen and fulvestrant-resistant ER+ BC patients. The drug induces apoptosis along with inhibiting metastasis. The dual function is performed by initiating epigenetic changes on CDK4/6 which leads to its repression and histone3 deacetylation on position lysine 27. The treatment efficacy can be increased by combining NM treatment with CDK4/6 inhibitor [29]. NM shows fewer side effects. However, it is used in acute pancreatitis, and in some patients shows allergic, mostly anaphylactic, reactions [33].

## 7. The scope of the drug-resistant study in genetic variations in ER cancer types

Clinical outcomes for BC patients with the same diagnostic and clinical prognostic profile might vary significantly. Our existing taxonomy of breast tumours, which mostly bases clinical classifications on appearance rather than molecular distinction, could be blamed for this discrepancy. With its capacity to concurrently investigate 10 000–40 000 genes, microarray technology has altered how we think about the molecular categorization of human malignancies [36]. Gene expression signatures or profiling (GEP) tests have been developed over the past 20 years for patients with luminal BC to predict the risk of disease recurrence and determine the possible benefit of undergoing adjuvant systemic chemotherapy.

BC is widely acknowledged as a condition with unique clinical characteristics and molecular characteristics, particularly ER+ and ER− subtypes [38]. The creation of various new high-throughput analytical techniques has lately made it feasible to achieve an aim known as precise molecular characterization or ‘fingerprint’ of cancer. This comprises methods for DNA, mRNA and protein analyses within a cell [150]. Researchers have posited that different genes are associated with treatment response in ER+ and ER− cancer because of the large genetic variation between ER+ and ER− tumours, as indicated by a meta-analysis of BC patient tumour samples [151]. Shen *et al*. [36], to a considerable degree, identified genes whose expression is associated with responsiveness to chemotherapy drugs, particularly several chemotherapeutic agents, using human BC ER+ and ER− cell lines [36]. The findings demonstrate that ER+ cell lines have different multidrug response genes than ER− cell lines. Additionally, functional research shows that distinct biological processes in ER+ versus ER− breast cells are connected to medication responsiveness. The majority of the enriched pathways found in ER+ cells are linked to different forms of cellular signalling, such as cell cycle control, apoptosis, cellular stress and damage, cytokine signalling and growth factor signalling [36].

Future studies using the full transcriptome could contribute to the development of even more sophisticated classifiers as both coding and non-coding gene variations have an impact on how various BC molecular subtypes behave [152]. A recent study exploring the growing need to improve existing molecular techniques and incorporate Tumour Microenvironment (TME) traits, especially in BC patient groups with worse prognoses, including ER/HER2 and HER2+ patients also concluded that the field of gene expression-based forecasting models is growing, supported by the clinical efficacy of these multigenic tests which has been validated and is robust, with realized benefits [153].

## 8. Discussion

BC is among the most occurring cancers in women, estimated to be around 24.5% of total cancer affecting females. Variation in ER expression is a major factor in the development of hormone-dependent BC [4]. Endocrine treatments are the conventional methods used to treat BC which include SERMS and AIs [13].

Tamoxifen, a SERM, inhibits the E2-mediated action of AF2, causing it to become ER-antagonistic while still retaining some partial agonistic effect [17]. The inhibition of oestrogen-regulated genes causes a block in the cell cycle’s G1 phase and ultimately leads to programmed cell death [24,25]. Toremifene is also a SERM, which is a chlorinated derivative of tamoxifen [42]. Its functioning is like tamoxifen with the benefit of lower side effects in comparison to tamoxifen [41]. Raloxifene is also a SERM that can function as both agonistic or antagonistic depending on its action on co-activators and co-repressors that are attracted to the ER target gene [31,32]. Another conventional drug used to treat BC is fulvestrant. It is a SERD which inhibits ER dimerization by binding to its monomer units [52]. This leads to the downregulation of ER functioning as fulvestrant binding causing monomer degradation and an ERα level decrease [35]. Letrozole is an AI, which hinders aromatase activity by binding to cytochrome P450. This mechanism causes inhibition of oestrogen synthesis preventing tumour growth and promoting its regression [37,57].

However, it was observed that the effectiveness of these drugs was hindered owing to inherited or acquired resistance. Resistance may occur owing to genetic mutations, epigenetic variations as well as miRNA level variations. The genetic variations leading to drug resistance have been observed in ABC transporter genes, AKT gene, *S100A8/A9* gene and *TAGLN2*. Alteration in the *ABCB1* gene causes paclitaxel and anthracycline resistance and ABCG2 gene variation causes doxorubicin, mitoxantrone and tivantinib resistance [75,76,79,80]. These are owing to the increased drug efflux by the mutated ABC transporter [61,62]. Mutational change in AKT overactivates it and causes continuous activation of the PI3K/AKT/mTOR pathway contributing to cell proliferation and other cancer cell properties [63,64,82]. This is reported in paclitaxel resistance in TNBC patients [81,83]. *S100A8/A9* gene alteration is also observed in tamoxifen resistance [89]. Transgelin-2 (*TAGLN2*) gene alteration leads to overexpression of the protein which accelerates ABC transporter expression and deregulates PTEN expression which is seen in paclitaxel resistance. This not only increases drug efflux but also activates the PI3K/AKT/GSK-3β pathway in the absence of PTEN and promotes tumour growth [90,91].

miRNA also contributes to drug resistance, like fulvestrant resistance, owing to *miR-33a* expression, nischarin resistance owing to *miRNA-27b* downregulation, doxorubicin resistance owing to miR-298 upregulation as well as IncRNA-HOTAIR expression [67,98,102,105,112]. Also, *miRNA-3130* and *miRNA-548* expression contribute to MDR. The miRNA expression causes upregulation or downregulation of gene activity or participates in cell signalling that causes tumour development properties. Genes are exposed to epigenetic changes that regulate gene expression. The irregular epigenetic changes cause cells to become cancerous. *DOK7* hypermethylation and *CTCF* hypermethylation are found to give rise to tamoxifen-resistant BC [114,118]. Histone3 acetylation causes doxorubicin resistance owing to the loss-of-function of MMR and also causes MDR if the acetylation occurs at the lysine residue of the protein [69,154].

Though BC is mainly subgrouped and the meta-analysis is done as ER+ or ER−, the latest research is now incorporating the TME trait study to analyse the cancerous cells better [38,151,153]. The new method will further group BC into a more specific subgroups to improve clinical efficacy [153]. Other than the TME method, NGS is a potential method to detect the discussed alteration and help to provide personalized treatment at an early stage of detection. ChIP is an existing method used to detect epigenetic variation, especially, histone modification. In addition, novel techniques have evolved like dPCR to detect genomic mutations and electrochemical biosensors to detect miRNA variations (especially found in BC patients). To treat drug-resistant BC, novel therapies are being researched using various cell lines. One of the latest researched techniques is PDT which uses photosensitizers to target ABC transporters, to decrease drug efflux and increase endocrine drug efficacy [105,135]. The therapy is being researched for improvement owing to lower efficacy in glutathione-rich tumour environments. It is combined with a mitochondrial complex III inhibitor which causes glutathione synthesis inhibition and better PDT efficacy in TNBC cells [137,138]. Guajadial and talazoparib are novel drugs used to treat drug-resistant BC by targeting genes of ABC transporters [140,141]. Talazoparib is being researched to be used in daunorubicin- and mitoxantrone-resistant cancer cells [141]. The FDA in the USA has already approved the drug to treat Breast Cancer *A* gene (*BRCA*)-mutated and HER2-negative BC. For drug resistance caused by miRNA expressional changes, miRNA inhibitors or activators can be used, like miR-1246 inhibitors in docetaxel-resistant cells as researched by Bott e*t al*. [147]. Cannabidiol showed positive results on tamoxifen-resistant cell lines by inhibiting *CYP3A4* and *CYP2D6*, decreasing tamoxifen metabolism and hence increasing its efficacy [148,149]. Also, FA-17 and nafamostat mesylate are being researched for endocrine-resistant drug resistance.

## 9. Conclusion

BC treatment including SERMs, SERDs and others have been effective for a long time, but owing to genetic variations like mutational changes, epigenetic changes and miRNA alterations, the target protein of these therapies may be altered. This results in failure of drug action or low efficacy, giving rise to drug-resistant BC. As discussed in this review, genes like *ABCB1, CYP2D6, S100A8/A9, TAGLN2* and *NPM* are observed to be altered. Other than these genes, miRNA level alteration, like miR-33a, -27b, miR-298, miR-1246, miR-3130 and miR-584, and epigenetic alteration, like *CTCF* hypermethylation and histone3 acetylation, led to drug resistance in BC patients. These can be detected by methods such as NGS, dPCR, ChIP and electrochemical biosensors. These findings have enabled researchers to explore these molecules as a novel therapeutic target. PDT is a novel therapy researched to be used in combination with BC treatment; tamoxifen, toremifene and with mitochondrial complex III inhibitors to treat MDR by reversing the mutational protein effect on endocrine drugs. Guajadial and talazoparib are novel drugs, used to treat drug-resistant BC by targeting genes of ABC transporters. COX2 along with DOX is also a potent combinational therapy to control excess drug efflux by P-gp and better efficacy of DOX in MDR BC patients. FA-17, cannabidiol and other compounds are under ongoing research as potent drugs for drug-resistant BC, especially in tamoxifen and fulvestrant resistance. Knowledge of the alteration can be a boon for further development of therapeutic as well as diagnostic methods. This will help to accelerate personalized medication research to gain better and faster treatment for BC patients.

## Data Availability

All data analysed are included within this article.
